# Introducing shrinkage in heavy-tailed state space models to predict equity excess returns

**DOI:** 10.1007/s00181-023-02437-3

**Published:** 2023-05-29

**Authors:** Florian Huber, Gregor Kastner, Michael Pfarrhofer

**Affiliations:** 1 Department of Economics, University of Salzburg, Salzburg, Austria; 2 Department of Statistics, University of Klagenfurt, Universitätsstraße 65-67, 9020 Klagenfurt, Austria; 3 Department of Economics, University of Vienna & WU Vienna, Vienna, Austria

**Keywords:** S$\&~$P 500, Fundamental factors, Dynamic regression, Stochastic volatility, Non-Gaussian models, G12, C11

## Abstract

We forecast excess returns of the S &P 500 index using a flexible Bayesian econometric state space model with non-Gaussian features at several levels. More precisely, we control for overparameterization via global–local shrinkage priors on the state innovation variances as well as the time-invariant part of the state space model. The shrinkage priors are complemented by heavy tailed state innovations that cater for potential large breaks in the latent states, even if the degree of shrinkage introduced is high. Moreover, we allow for leptokurtic stochastic volatility in the observation equation. The empirical findings indicate that several variants of the proposed approach outperform typical competitors frequently used in the literature, both in terms of point and density forecasts.

## Introduction

In this paper, we propose a flexible dynamic regression model with time-varying parameters (TVPs). Such models are prone to overfitting, and we introduce a global–local shrinkage prior to regularize the potentially high-dimensional parameter space. In addition, we consider non-Gaussian error terms in both the state and measurement equations, which links our proposed framework to recent contributions on mixture innovation models and time-varying shrinkage processes. We apply our model for predicting monthly equity premia using the S &P 500 index and a set of relevant market fundamentals. This connects our paper to the literature on modeling aggregate portfolio returns using key macroeconomic and financial predictors (see also Welch and Goyal 2008).

A generic version of our proposed framework posits a time-varying relationship between a scalar dependent $y_t$ (excess returns in our application) and a set of *K* predictors in ${\varvec{X}}_t$ (e.g., market fundamentals or macroeconomic quantities), given by the dynamic regression model:

1.1 $$\begin{aligned} y_t&= {\varvec{\beta }}'_t {\varvec{X}}_t + \varepsilon _t, \end{aligned}$$

1.2 $$\begin{aligned} {\varvec{\beta }}_t&={\varvec{\beta }}_{t-1}+{\varvec{w}}_t, \end{aligned}$$

for $t = 1,\dots , T$, see, e.g., West and Harrison (2006). Here, it is assumed that the regressors are related to $y_t$ through a set of *K* dynamic (time-varying) regression coefficients ${\varvec{\beta }}_t$ that follow a random walk process with ${\varvec{w}}_t \sim \mathcal {N}({\varvec{0}}_K, {\varvec{V}})$, where ${\varvec{V}}=\text {diag}(v_1, \dots , v_K)$ is a diagonal variance-covariance matrix of dimension $K\times K$. To simplify computation, the measurement errors captured through $\varepsilon _t$ are often assumed to follow a zero mean Gaussian distribution with variance $\sigma ^2_\varepsilon$. In what follows, we relax the assumption of Gaussianity for both $\varepsilon _t$ and ${\varvec{w}}_t$ and introduce a heavy-tailed error distribution for these innovations.

Model specification within this baseline econometric framework has received considerable attention recently (see, among many others, Frühwirth-Schnatter and Wagner 2010; Eisenstat et al. 2016; Bitto and Frühwirth-Schnatter 2019; Huber et al. 2021; Hauzenberger et al. 2021). One prevalent issue is that, if left unrestricted, Eq. (1.1) has a strong tendency to overfit the data, leading to imprecise out-of-sample forecasts. This calls for some form of regularization. Frühwirth-Schnatter and Wagner (2010) show how a non-centered parameterization of the state space model can be used to apply a standard Bayesian shrinkage prior on the process variances in ${\varvec{V}}$. This approach allows for capturing model uncertainty along two dimensions: The first dimension is whether a given element in ${\varvec{X}}_t$, $X_{jt}$, should be included or excluded. The second dimension addresses the question whether the associated element in ${\varvec{\beta }}_t$, $\beta _{jt}$, should be constant or time varying. Note that the latter is equivalent to setting $v_j=0$, which yields $\beta _{jt}=\beta _{jt-1}$ for all *t*.

In the present contribution, we combine the literature on shrinkage and variable selection within the general class of state space models (Frühwirth-Schnatter and Wagner 2010; Eisenstat et al. 2016; Bitto and Frühwirth-Schnatter 2019) with the literature on non-Gaussian state space models (Carlin et al. 1992; Kitagawa 1996). The model we propose features *t*-distributed shocks to both the observation and the state equation. This choice provides enough flexibility to capture large outliers commonly observed in stock markets. To cope with model and specification uncertainty, we adopt the Dirichlet–Laplace (DL, Bhattacharya et al. 2015) shrinkage prior that allows for flexible shrinkage toward simpler nested model specifications. This prior has been used for macroeconomic and financial data in, e.g., Cross et al. (2020) and Koop et al. (2022), most recently. One key empirical observation from the macroeconomic literature (Sims and Zha 2006; Koop et al. 2009) is that parameters may exhibit periods of smooth state evolution, abrupt breaks or no time variation at all (see also Hauzenberger 2021). We capture this stylized fact by assuming that the shocks to the states follow a (potentially) heavy-tailed *t*-distribution that allows for large jumps in the regression coefficients, even in the presence of strong shrinkage toward constancy.

To assess the merits of the novel econometric features, we apply several nested variants of this model to S &P 500 index data, revisiting questions about the predictability of excess returns. Predicting equity prices has been one of the main challenges for financial economists during the last decades. A plethora of studies emerged that draw a relationship between different macroeconomic and financial fundamentals and the predictability of excess returns (Lettau and Ludvigson 2001; Ang and Bekaert 2007; Welch and Goyal 2008; Dangl and Halling 2012, among others).^1^ While some authors find evidence of predictability, simple naive benchmarks still prove to be extremely difficult to beat by more sophisticated models.

To investigate whether these econometric extensions also translate into predictive gains, we apply our proposed model framework to the well-known dataset compiled in Welch and Goyal (2008). More specifically, we forecast monthly S &P 500 excess returns over a period of 55 years and compute one-step-ahead predictive densities, relating our application to cliometrics.^2^ We then assess to what extent the proposed methods outperform simpler nested alternatives and other competing approaches both in terms of root mean square forecast errors (RMSEs) and log predictive scores (LPSs). Having such a long sample of observations has several advantages, such as being able to assess slow/fast moving variation in parameters across many different economic periods and phases. In addition, based on the close relationship between LPSs and marginal likelihoods, it provides us with a robust measure of model fit (see also Geweke and Amisano 2010).

Our results indicate that a time-varying parameter model with shrinkage and heavy-tailed measurement errors displays the best predictive performance over the full hold-out period. Considering the results within expansions and recessions highlights that allowing for heavy-tailed state innovations pays off in economic downturns, while it is outperformed by a specification with heavy tailed measurement errors in expansions. A dynamic model selection exercise shows that forecasting performance may be further improved by computing model weights based on previous predictive likelihoods. Strong overall forecasts generally translate into a favorable performance in terms of Sharpe ratios. Using this economic evaluation criterion suggests that models that work well in forecasting also work well when used to generate trading signals.

The remainder of the paper is structured as follows. Section 2 introduces the necessary modifications to the econometric model postulated in Eqs. (1.1) and (1.2) to allow for heavy-tailed measurement and state innovations. In addition, the section provides an overview on the Bayesian prior setup. Section 3 presents the empirical findings, focusing on the results of our forecasting exercise. Finally, the last section summarizes and concludes the paper.

## Econometric framework

### A non-Gaussian state space model

The shocks to both the measurements and the states are assumed to follow Gaussian distributions with constant variances in Sect. 1. For financial data, this could be overly restrictive, and especially, the assumption of homoskedasticity is likely to translate into weak predictive performance. Such non-Gaussian features may be even stronger for disaggregated or higher-frequency data, but, as the results in Sect. 3 show, are clearly present for the monthly S &P 500 index returns.

As a remedy, we propose the measurement errors to follow a *t*-distribution with $\nu$ degrees of freedom and time-varying variance,

2.1 $$\begin{aligned} \varepsilon _t|h_t, \nu&\sim {t}_\nu (0, e^{h_t}), \end{aligned}$$

2.2 $$\begin{aligned} h_t|h_{t-1}&\sim \mathcal {N}(\mu + \rho (h_{t-1}-\mu ), \sigma ^2_h),\end{aligned}$$

2.3 $$\begin{aligned} h_0&\sim \mathcal {N}\left( \mu , \sigma _h^2/(1-\rho ^2)\right) , \end{aligned}$$

where $\mu$ denotes the unconditional mean of the log-volatility process $h_t$, $\rho$ its autoregressive parameter and $\sigma ^2_h$ its innovation variance. Introducing auxiliary variables ${\varvec{\tau }} = (\tau _{1}, \dots , \tau _T)'$ permits stating Eq. (2.1) as a conditional Gaussian distribution,

2.4 $$\begin{aligned} \varepsilon _t|h_t,\tau _{t}&\sim \mathcal {N}(0, \tau _{t} e^{h_t}), \end{aligned}$$

2.5 $$\begin{aligned} \tau _t&\sim \mathcal {G}^{-1}(\nu /2, \nu /2), \end{aligned}$$

where $\mathcal {G}^{-1}$ refers to an inverse Gamma distribution. This specification of the measurement errors allows capturing large shocks as well as time variation in the underlying error variances. Especially for financial data that are characterized by heavy-tailed shock distributions as well as heteroskedasticity, this proves to be a key feature to produce precise predictive densities. Furthermore, we assume that the shocks to the latent states follow a heavy-tailed error distribution. Similar to Eqs. (2.1) and (2.4), the state innovations follow a *t*-distribution with $\kappa _j$ degrees of freedom,

2.6 $$\begin{aligned} w_{jt}|\kappa _j \sim t_{\kappa _j}(0, v_j)\quad \Leftrightarrow \quad w_{jt}|\xi _{jt} \sim \mathcal {N}(0, \xi _{jt} v_j), \end{aligned}$$

where the elements of ${\varvec{\xi }}_j = (\xi _{j1},\dots ,\xi _{jT})$ follow independent $\mathcal {G}^{-1}(\kappa _j/2, \kappa _j/2)$ distributions. In contrast with Eq. (2.1), we assume that the shocks to the states are homoskedastic. Notice that Eq. (2.6) effectively implies that we occasionally expect larger breaks in the underlying regression coefficients, even if $v_j$ is close to zero. This appears to be of particular importance when shrinkage priors are placed on $v_j$.

### A Dirichlet–Laplace shrinkage prior

The model described in the previous sections is heavily parameterized and calls for some sort of regularization in order to provide robust and accurate forecasts. To this end, we follow Frühwirth-Schnatter and Wagner (2010) and exploit the non-centered parameterization of the model,

2.7 $$\begin{aligned} y_t =&{\varvec{\beta }}_0' {\varvec{X}}_t + \tilde{{\varvec{\beta }}}'_t \sqrt{{\varvec{V}}} {\varvec{Z}}_t + \varepsilon _t, \quad \varepsilon _t \sim {t}_\nu (0, e^{h_t}), \end{aligned}$$

2.8 $$\begin{aligned} \tilde{{\varvec{\beta }}}_t =&\tilde{{\varvec{\beta }}}_{t-1} + {\varvec{\eta }}_t,\quad {\varvec{\eta }}_t \sim \mathcal {N}({\varvec{0}}_K, {\varvec{I}}_K), \end{aligned}$$

2.9 $$\begin{aligned} {\beta }_{jt} =&{\beta }_{jt-1}+{w}_{jt}, \quad {w}_{jt}\sim t_{\kappa _j}(0, v_j), \text { for }j=1,\hdots ,K, \end{aligned}$$

where the last equation refers to the dynamic evolution of the non-normalized states. The *j*th element of $\tilde{{\varvec{\beta }}}_t$ is given by $\tilde{\beta }_{jt}=\frac{\beta _{jt}-\beta _{j0}}{\xi _{jt}\sqrt{v_{j}}}$, ${\varvec{V}} = \sqrt{{\varvec{V}}} \sqrt{{\varvec{V}}}$, and ${\varvec{Z}}_t$ is a *K*-dimensional vector with *j*th element given by $Z_{jt} = \sqrt{\xi _{jt}} X_{jt}$. For identification, we set $\tilde{{\varvec{\beta }}}_0 = {\varvec{0}}$. Notice that Eq. (2.7) implies that the process innovation variances as well as the auxiliary variables are transformed from the state to the observation equation. We exploit this by estimating the elements of ${\varvec{\beta }}_0$ and $\sqrt{{\varvec{V}}}$ through a standard Bayesian regression model.^3^

We use a Dirichlet-Laplace shrinkage prior (Bhattacharya et al. 2015) on ${\varvec{\alpha }}= ({\varvec{\beta }}_0', \sqrt{v_1}, \dots , \sqrt{v_K})'$. More specifically, for each of the 2*K* elements of ${\varvec{\alpha }}$, denoted by $\alpha _j$, we impose a hierarchical Gaussian prior given by

2.10 $$\begin{aligned} \alpha _j \sim \mathcal {N}(0, \psi _j \phi ^2_j \lambda ^2), \quad \psi _j \sim \textrm{Exp}(1/2), \quad \phi _j \sim \textrm{Dir}(a, \dots , a), \quad \lambda \sim \mathcal {G}(2 K a, 1/2). \end{aligned}$$

Here, $\psi _j$ denotes a local scaling parameter that is equipped with an exponentially distributed prior and ${\varvec{\phi }}= (\phi _1, \dots , \phi _{2K})$ is a vector of additional scaling parameters that are restricted to the $(2K-1)$-dimensional simplex, i.e., $\phi _j>0$ for all *j* and $\sum _{j=1}^{2K} \phi _j = 1$. For each $\phi _j$, we assume a symmetric Dirichlet distribution with intensity parameter *a* which we set to $a=1/(2K)$ in the empirical application.^4^ Finally, we let $\lambda$ denote a global shrinkage parameter that pulls all elements in ${\varvec{\alpha }}$ to zero. Due to the importance of this scaling parameter, we do not fix it a priori but impose a Gamma hyperprior and subsequently infer it from the data.

**Fig. 1:**
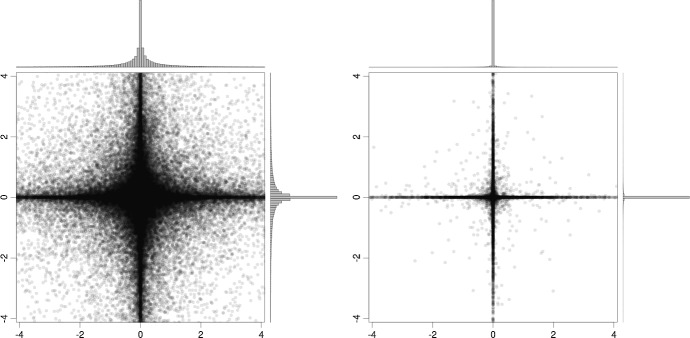
Scatterplots and histograms of $100\,000$ draws from the first two components of a DL(1/(2*K*)) prior for $K = 1$ (left) and $K = 13$ (right)

This prior setup has been shown to perform well for different models and applications (see also Li and Pati 2017; Feldkircher et al. 2017; Kastner and Huber 2020). Intuitively, it mimics the behavior of a point mass mixture prior but with the main advantage of computational tractability in high dimensions. The underlying marginal priors on $\alpha _j$ are all heavy-tailed, implying that even in the presence of a small global shrinkage parameter $\lambda$, we still allow for non-zero elements in ${\varvec{\alpha }}$. This feature has been identified to be crucial for good forecasting performance and, in addition, does well in discriminating signals from noise. In Fig. 1, the first two components of this prior are visualized for a univariate ($K = 1$) and a multivariate dynamic regression setting with $K = 13$ as in the empirical study in Sect. 3. Note that the marginal shrinkage effect becomes stronger with increasing *K*, while the kurtosis remains relatively stable.

This prior introduces shrinkage on the square root of the process innovation variances. Thus, we effectively assess whether coefficients are constant or time varying within a unified modeling framework.^5^ One key advantage of our model, however, is that the heavy-tailed innovations allow for breaks in the parameters even if the corresponding process innovation variances are close to zero. Thus, our framework is able to mimic models that only assume a small number of breaks in the regression coefficients, if necessary.

For the remaining coefficients, we follow Kim et al. (1998) and Kastner and Frühwirth-Schnatter (2014) and use a mildly informative Gaussian prior on the level of log variance, $\mu \sim \mathcal {N}(0, 10^2)$. On the (transformed) persistence parameter, we use a Beta prior $\frac{\rho +1}{2} \sim \mathcal {B}(25, 5)$, and on $\sigma ^2_h$ we use a Gamma prior, $\sigma ^2_h \sim \mathcal {G}(1/2, 1/2)$. Finally, on the degrees of freedom $\nu$ and $\kappa _j$ we impose independent $\mathcal {G}(1, 1/10)$ priors implying that both the prior means and the prior standard deviations are equal to 10.^6^ Details about the sampling algorithm are provided in Appendix A.

## Empirical application

In this section, we start by providing information on the data and model specification in Sect. 3.1. We proceed by describing the forecasting design and the set of competing models in Sect. 3.2, and present the main forecasting results in Sect. 3.3. The key empirical findings of our model for the full sample period can be found in Appendix B.

### Data overview and model specification

We adopt the dataset utilized in Welch and Goyal (2008) and establish a relationship between S &P 500 excess returns and a set of fundamental factors that are commonly used in the literature. Our dataset is monthly and spans the period from 1927:01 to 2010:12.^7^ The response variable is the S &P 500 index return minus the risk-free rate (treasury bill rate).

The following lagged explanatory variables are included in our models: The dividend price ratio (DP), the dividend yield (DY), the earnings price ratio (EP), the stock variance (SVAR, defined as the sum of squared S &P 500 daily returns), and the book-to-market ratio (BM). Furthermore, we include the ratio of 12-month moving sums of net issues by stocks listed on the New York Stock Exchange (NYSE) divided by the total end-of-year market capitalization of NYSE stocks (NTIS). Moreover, the models feature yields on short- and long-term government debt and information on term spreads (TBL, LTY and LTR). To capture corporate bond market dynamics, we rely on the spread differences between BAA and AAA rated corporate bond yields and the differences of corporate and treasury bond returns at the long end of the yield curve (DFY and DFR). Finally, the set of covariates is completed by consumer price inflation (INFL) and an intercept term (cons). For more information on the construction of the exogenous variables that mainly capture stock characteristics, see Welch and Goyal (2008).

We present selected full sample period results, illustrating the merits of the proposed approach, in Appendix B, and proceed in the main text by discussing the design of the forecasting exercise and introduce a set of competing models for forecasting excess returns.

### Design of the forecasting exercise and competitors

We utilize a recursive forecasting design and specify the period ranging from 1927:01 to 1956:12 as an initial estimation period. We then perpetually expand the initial estimation sample by one month until the end of the sample (2010:12) is reached. This yields a sequence of 647 monthly one-step-ahead predictive densities for S &P 500 excess returns, where we focus attention on root mean square forecast errors (RMSEs) and log predictive scores (LPSs, see Geweke and Amisano 2010, for a discussion) to evaluate the predictive capabilities of the model. Compared to the existing literature (Lettau and Ludvigson 2001; Ang and Bekaert 2007; Welch and Goyal 2008; Dangl and Halling 2012), this implies that we do not focus on point predictions exclusively but rely on a more general measure that takes into account higher moments of the corresponding predictive densities.

Our set of competing models includes the historical mean with stochastic volatility (labeled Mean-SV). This model, a strong benchmark in the literature, enables us to evaluate whether the inclusion of additional explanatory variables improves forecasting. Moreover, we also include a constant parameter regression model with SV (referred to as Reg-SV), a recursive regression model (labeled Recursive), an autoregressive model of order one with SV (AR(1)-SV), a random walk without drift and with SV (RW-SV), and the mixture innovation model proposed in Huber et al. (2019) featuring thresholded time-varying parameters (denoted TTVP). Moreover, to investigate which of the multiple features of our proposed model improve predictive capabilities, we include several nested versions: A time-varying parameter regression model with stochastic volatility and Gaussian shocks to both the measurement and the state equations with a DL shrinkage prior (labeled TVP-SV DL), a model that features *t*-distributed measurement errors (but Gaussian state innovations) and a DL prior (labeled t-TVP-SV DL 1), a specification that features *t*-distributed state innovations (but Gaussian measurement errors) and a DL prior (t-TVP-SV DL 2), and finally, the version of our proposed framework that features *t*-distributed state innovations and *t*-distributed measurement errors on top of the DL prior (t-TVP-SV DL 3).

A recent strand of the literature suggests that forecasts may be improved by selecting best-performing specifications dynamically from a pool of models, based on their past predictive performance (Raftery et al. 2010; Koop and Korobilis 2012; Onorante and Raftery 2016). Such methods involve computing a set of weights $\mathfrak {w}_{t|t-1,m}$ at time *t*, conditional on information up to $t-1$ for each model *m* within the model space $\mathcal {M}$. Specifically, we construct the weights as

3.1 $$\begin{aligned} \mathfrak {w}_{t|t-1,m} = \frac{\mathfrak {w}_{t-1|t-1,m}^\gamma }{\sum _{m\in \mathcal {M}}\mathfrak {w}_{t-1|t-1,m}^\gamma }, \quad \mathfrak {w}_{t-1|t-1,m} = \frac{\mathfrak {w}_{t-1|t-2,m}\times p_{t-1|t-2,m}}{\sum _{m\in \mathcal {M}}\mathfrak {w}_{t-1|t-2,m}\times p_{t-1|t-2,m}}. \end{aligned}$$

Here, $p_{t-1|t-2,m}$ is the one-step ahead predictive likelihood, and the parameter $\gamma =0.99$ imposes persistence in the model weights over time. This parameter is a forgetting factor with values close to one yields a specification that takes into account also the less recent forecast performance. The initial model weights are assumed to be equal across all models. To choose the model per period, we select the one with the highest weight $\mathfrak {w}_{t|t-1,m}$ and label this approach dynamic model selection (DMS) in subsequent discussions.

### Predicting the US equity premium

Table 1 displays relative RMSEs and differences in log predictive scores relative to the Mean-SV benchmark. For relative RMSEs, numbers exceeding unity indicate outperformance of the benchmark model, whereas numbers smaller than one indicate a stronger performance of the model under consideration. For the relative LPSs, a positive number indicates that a given model outperforms the benchmark model. We focus attention on forecasting accuracy during distinct stages of the business cycle (i.e. recessions/expansions), dated by the NBER Business Cycle Dating Committee.^8^ In doing so, we can investigate whether model performance changes over business cycle stages. Finally, we also report results over the full sample period.

**Table 1:** Root mean square errors and log predictive scores relative to the historical mean with SV model

	Relative root mean square errors			Log Bayes factors		
	Recession	Expansion	Full sample	Recession	Expansion	Full sample
Recursive	0.914	0.945	0.933	$-$4.888	$-$134.470	$-$139.358
Reg-SV	0.933	0.982	0.964	6.703	5.165	11.869
RW-SV	1.006	1.000	1.000	$-$0.426	$-$3.124	$-$3.550
AR(1)-SV	0.970	0.969	0.968	3.124	7.315	10.439
TVP-SV DL	0.918	0.955	0.941	8.900	8.205	17.105
t-TVP-SV DL (1)	0.923	0.955	0.943	8.416	9.627	18.043
t-TVP-SV DL (2)	0.922	0.953	0.941	8.716	8.938	17.654
t-TVP-SV DL (3)	0.929	0.960	0.948	8.103	3.231	11.334
TTVP	0.945	0.966	0.957	6.074	8.293	14.367
DMS	0.925	0.955	0.944	8.170	12.066	20.236

We start by considering point forecasting performance before turning to density forecasts. The left panel of Table 1 suggests that most specifications considered improve upon the Mean-SV benchmark over the full sample, as well as during recessionary and expansionary episodes. We find that the t-TVP-SV specifications with a DL prior all perform rather well, outperforming the benchmark up to over eight percent during recessions (in the case of the TVP-SV DL) and up to 5.7 percent over the full sample. It is noteworthy that constant parameter models, while outperforming the no-predictability benchmark, only yield small gains in predictive accuracy and this result confirms findings in Welch and Goyal (2008) and Dangl and Halling (2012). The DMS point forecasts are rather close to those of the time-varying parameter specifications.

One key finding is that accuracy improvements in recessions tend to be more pronounced, indicating that using more information seems to pay off during economic downturns. We conjecture that larger information sets contain additional information necessary to better predict directional movements and this, in turn, improves point forecasting performance. Considering the results during expansions yields a similar picture: all state space models using some sort of shrinkage (including the TTVP specification) display a favorable point forecasting performance. While differences across models appear to be rather muted, this small premium in forecasting accuracy can be traced back to a feature attributed to the combination of shrinkage priors and heavy-tailed process innovations.

The discussion above focused on point forecasts exclusively. To additionally assess how well the models perform in terms of density forecasting, the right panel of Table 1 presents relative LPSs. A few results are worth emphasizing. First, dynamically selecting the best performing model over time based on past predictive likelihoods pays off and yields superior performance in terms of density forecasts for the full sample and expansions. Second, focusing on individual specifications over the model space, the last column of Table 1 reveals that most models under consideration outperform the historical mean model with SV by large margins over the full sample. This finding can be traced back to the fact that the Mean-SV includes no additional covariates, and is thus unable to explain important features of the data that are effectively picked up by having additional exogenous covariates. Considering the forecast differences across models shows that introducing shrinkage in the TVP regression framework seems to pay off. Notice, however, that in terms of predictive capabilities, it suffices to allow for fat tailed innovations in either the state or measurement errors. Allowing for *t*-distributed errors for the shocks in the state and the observation equation generally yields weaker forecasting performance. A closer look at the underlying predictive density reveals that the predictive variance in that case appears to be slightly overestimated relative to the simpler specifications.

Second, zooming into the results for distinct stages of the business cycles indicates that t-TVP-SV DL 2 outperforms all competing model specifications during recessions. Especially when benchmarked against a simple random walk and the historical mean model, we find sharp increases in predictive accuracy when the more sophisticated approach is adopted. Considering the results for a constant parameter regression model also points toward favorable predictive characteristics of this simple specification in terms of density predictions. As in the case of point forecasts, we generally attest our models higher predictive capabilities during business cycle downturns and are thus in line with the recent literature (Rapach et al. 2010; Henkel et al. 2011; Dangl and Halling 2012).

This result, however, does not carry over to expansionary stages of the business cycle. The penultimate column of Table 1 clearly shows that while models that perform well during recessions also tend to do well in expansions, the single best performing model is the t-TVP-SV DL 1 specification. By contrast, the flexible t-TVP-SV DL 3 model performs poorly during expansions. This stems from the fact that equity price growth appears to be quite stable during expansions and thus corroborates the statement above: in expansions, this specification simply yields inflated credible intervals and thus weaker predictive density forecasting performance.

These findings suggest that the strong overall performance of t-TVP-SV DL 1 is mainly driven by superior forecasting capabilities during expansions, whereas this model is slightly outperformed by t-TVP-SV DL 2 during recessionary periods. During turbulent times, we find that controlling for heteroskedasticity is important, corroborating findings reported in the literature (Clark 2011; Clark and Ravazzolo 2015; Huber 2016; Kastner 2019). Moreover, the results also indicate that allowing for heavy-tailed shocks to the states helps to capture sudden shifts in the regression coefficients, a feature that appears to be especially important during recessions.

**Fig. 2:**
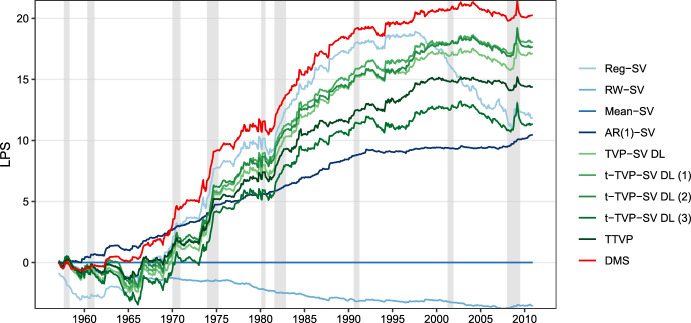
Log predictive Bayes factors relative to the historical mean model with stochastic volatility: 1957:01–2010:12. Gray shaded areas indicate NBER recessions. (Color figure online)

**Fig. 3:**
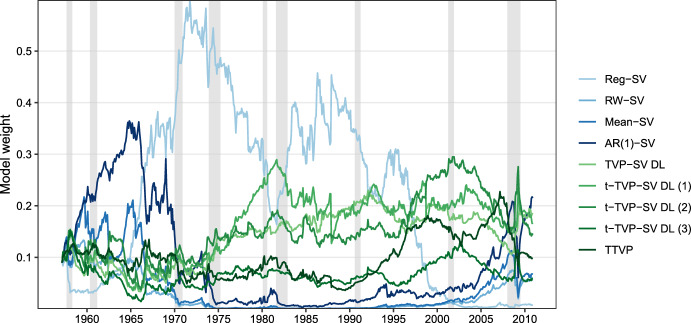
Dynamic model weights $\mathfrak {w}_{t|t-1,m}$ for model selection: 1957:01–2010:12. Gray shaded areas indicate NBER recessions. (Color figure online)

The previous discussion focused on overall forecast performance and highlighted that predictive accuracy depends on the prevailing economic regime. In crisis episodes, models that are generally quite flexible yield pronounced accuracy increases. Moreover, there is substantial evidence for predictive gains when dynamically selecting models. In the next step, we analyze whether there exists additional heterogeneity of forecast performance over time that is not specific to whether the economy is in a recession or expansion. To this end, Fig. 2 displays the evolution of the relative LPSs over time, and Fig. 3 relatedly indicates the underlying model weights for the DMS specification.

Figure 2 indicates that the DMS specification outperforms all other specifications for the most part of the holdout sample. This implies that the approach to calculating model weights appears to capture shifts in a model’s predictive performance quite well. After an initial period from the start of the holdout to the beginning of the 1970 s, the AR(1)-SV specification is the best performing model. From the midst of the 1970 s up to the midst of the 1990 s, a constant parameter model with SV outperformed all models considered. From around 1995 onwards, we observe a pronounced decline in forecasting performance of the Reg-SV specification over time, while all models that feature time-variation in their parameters produced a rather stable predictive performance. During the great financial crisis, all models except the RW-SV outperform the benchmark. This again highlights that, especially during crisis episodes, introducing shrinkage and time-varying parameters yields pronounced gains in forecast accuracy.

Given the specification of $\mathfrak {w}_{t|t-1,m}$ in Eq. (3.1) and the evolution of LPSs in Fig. 2, the findings for the model weights depicted over time in Fig. 3 are unsurprising. After an initial eight-year period where the proposed procedure dynamically selected the AR(1)-SV model, the dominating model until 1980 is the regression model with constant parameters and SV. Subsequently, for a brief period of approximately three years, t-TVP-SV-DL 1 received the largest model weight. Afterward, up to the mid/late 1990 s, the constant parameter model with SV, again, was selected as the best-performing model based on past predictive likelihoods. The pronounced decline in forecast performance discussed for Reg-SV in the context Fig. 2, however, also resulted in the model essentially receiving zero weight from 1995 onwards, where t-TVP-SV-DL 2 receives the highest weights in most cases.

**Fig. 4:**
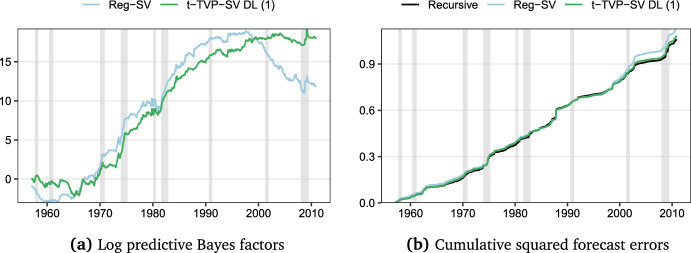
Log predictive Bayes factors relative to the historical mean model and cumulative squared forecast errors: Best performing models. Gray shaded areas indicate NBER recessions. (Color figure online)

In order to investigate where forecasting gains stem from, Fig. 4, left panel, displays the log predictive Bayes factors of Reg-SV and t-TVP-SV DL 1 relative to Mean-SV, whereas the right panel shows the cumulative squared forecast errors over the hold-out period. This figure clearly suggests that the sharp decline in predictive accuracy of the Reg-SV model mainly stems from larger forecast errors, as opposed to other features of the predictive density. The weaker point forecasting performance can be explained by the lack of time-variation in the parameters of the Reg-SV model. Notice that the recursive forecasting design implies that coefficients are allowed to vary over the hold-out period, but comparatively slower than under a time-varying parameter regression framework. Thus, while the coefficients in t-TVP-SV DL 1 are allowed to change rapidly if economic conditions change, the coefficients in Reg-SV take longer to adjust and this might be detrimental for predictive accuracy. For the sake of completeness, we also include the recursive regression in the right panel. An interesting finding is that homoskedastic errors appear to result in lower squared forecast errors, comparable to those of t-TVP-SV DL 1.

## Concluding remarks

This paper proposes a flexible econometric model that introduces shrinkage in the general state space modeling framework. We depart from the literature by assuming that the shocks to the state as well as observation are potentially non-Gaussian and follow a *t*-distribution. Assuming heavy-tailed measurement errors allows capturing outlying observations, while *t*-distributed errors in the state equation allow for large shocks to the latent states. This feature, in combination with a set of global–local shrinkage priors, allows for flexibly assessing whether time-variation is necessary and also, to a certain extent, mimics the behavior of models with a low number of potential regime shifts.

In the empirical application, we forecast S &P 500 excess returns. Using a panel of macroeconomic and financial fundamentals and a large set of competing models that are commonly used in the literature, we show that our proposed modeling framework yields sizeable gains in predictive accuracy, both in terms of point and density forecasting. We find that using the most flexible specification generally does not pay off relative to using a somewhat simpler specification that either assumes *t*-distributed shocks in the measurement errors or in the state innovations. Especially during economic downturns, we find that combining shrinkage with non-Gaussian features in the state equation yields strong point and density predictions, whereas in expansions, a model with *t*-distributed measurement errors performs best. This model also performs best if the full hold-out period is taken into consideration.

Our model has several limitations, and addressing these is something we leave open for further research. First, our approach is applied to a univariate response variable exclusively. Given that our flexible set of shrinkage priors effectively deal with overparameterization concerns, a natural extension of the framework would be to estimate a system of multiple financial quantities jointly in a VAR or use it to model time-varying covariance matrices. Second, in our empirical work, we have shown that parameters (within each group of covariates) co-move. This suggests that a factor structure on the coefficients along the lines suggested by Chan et al. (2020) or Fischer et al. (2023) could further improve predictive performance. Third, our shrinkage priors are static, ruling out dynamic shrinkage of the form suggested in Kowal et al. (2019) and applied to macroeconomic data in Huber and Pfarrhofer (2021).

## Figures and Tables

**Fig. 5:**
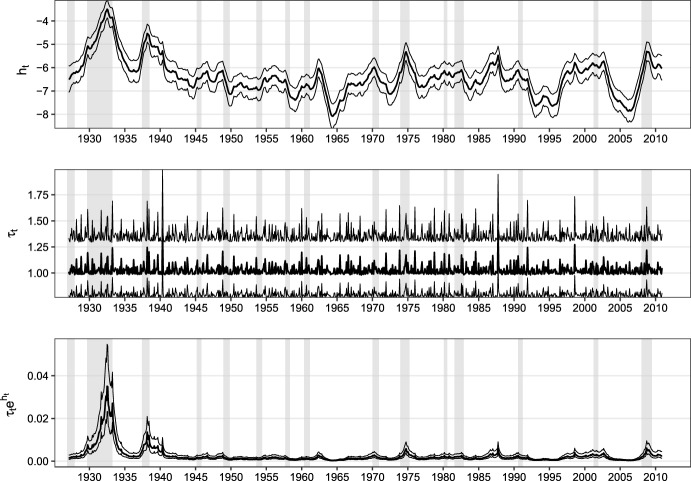
Volatility related components over time: 1927:01–2010:12. Gray shaded areas indicate NBER recessions. (Color figure online)

**Fig. 6:**
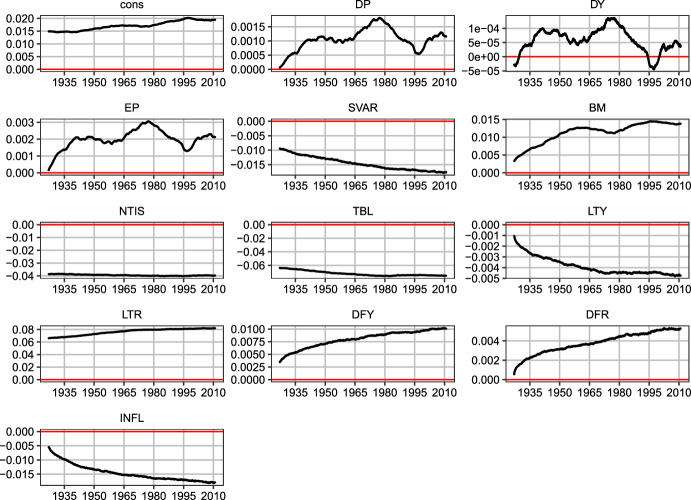
Time-varying regression coefficients: 1927:01–2010:12

## Appendix A: Full conditional posterior simulation

We carry out posterior inference using an MCMC algorithm that is repeated $30\,000$ times, with the first $15\,000$ draws discarded as burn-in. The full conditional posterior distributions all have well-known forms, and we can thus set up a Gibbs sampling algorithm that iteratively draws from all relevant distributions. Considered individually, each step has been discussed in previous papers, and we thus provide a brief summary:

- Conditional on the remaining parameters and states, we simulate the full history of $\tilde{{\varvec{\beta }}_t}$ for $t = 1,\dots ,T$ using a standard forward filtering backward sampling (FFBS) algorithm (Carter and Kohn 1994; Frühwirth-Schnatter 1994).
- ${\varvec{\beta }}_0$ as well as the diagonal elements of $\sqrt{{\varvec{V}}}$ are simulated from a Gaussian conditional posterior distribution by noting that Eq. (2.7) resembles a standard regression model with heteroskedastic shocks.
- The full conditional distribution of the local shrinkage parameters is inverse Gaussian, i.e., $\psi _j|\bullet \sim {iG}(\phi _j \lambda / |\alpha _j|, 1), \ j=1,\dots ,2K$. To draw from this distribution, we use the rejection sampler of Hörmann and Leydold (2013) via the R package GIGrvg (Leydold and Hörmann 2017).
- The global shrinkage parameter conditionally follows a generalized inverse Gaussian distribution, i.e., $\lambda |\bullet \sim \mathcal {GIG}\left( 2K(a - 1),1, 2\sum _{j=1}^{2K} |\alpha _j|/\phi _j\right)$, which is again easily accessible through GIGrvg.
- The scaling parameters $\phi _j$ are drawn by first sampling auxiliary quantities $T_j$ from $\mathcal {GIG}(a-1, 1, 2|\alpha _j|),$ and then setting $\phi _j = T_j/\sum _{i=1}^{2K} T_i$ which yields a draw from ${\varvec{\phi }}|{\varvec{\alpha }}$ (Bhattacharya et al. 2015).
- Each element of the auxiliary vector ${\varvec{\tau }}$ is conditionally inverse Gamma distributed, i.e., $\tau _t|\bullet \sim \mathcal {G}^{-1}\{(\nu + 1)/2,(\nu + \epsilon _t^2\exp (-h_t))/2\},$ independently for $t \in \{1, \dots , T\}$, which makes sampling from this distribution straightforward. Draws from ${\varvec{\xi }}_j|\bullet$ for all *j* are obtained analogously.
- The conditional likelihood for the degrees of freedom parameter $\nu$ reads
  A.1 $$\begin{aligned} p({\varvec{\tau }}|\nu ) \propto \left( \frac{\nu }{2}\right) ^{n\nu /2} \Gamma \!\left( \frac{\nu }{2}\right) ^{-n} \left( \prod _{t=1}^n \tau _t\right) ^{-\nu /2} \exp \left\{ -\frac{\nu }{2}\sum _{t=1}^n\frac{1}{\tau _t}\right\} \end{aligned}$$
  To obtain draws from the full conditional distribution, $\nu |\bullet = \nu |{\varvec{\tau }}$, we use an independence Metropolis–Hastings update in the spirit of Chib and Greenberg (1994). We find the maximizer of Eq. (A.1) and the corresponding Fisher information, which we, in turn, use to construct a Gaussian proposal distribution. For details, see Kastner (2015); for alternatives, see Hosszejni and Kastner (2021). Draws from $\kappa _j|\bullet$ for all *j* are obtained analogously.
- Conditional on all other parameters, updating the latent log variances ${\varvec{h}} = (h_0, h_1, \dots , h_T)$ and the stochastic volatility parameters $\mu$, $\rho$, and $\sigma _h^2$ is done exactly as in Kastner and Frühwirth-Schnatter (2014), who utilize an efficient auxiliary mixture sampler (Omori et al. 2007) with ancillarity–sufficiency interweaving (ASIS, Yu and Meng 2011). We access this sampler through the implementation in the R package stochvol (Kastner 2016).

## Appendix B: Empirical results for the full sample period

In this appendix, we use our proposed non-Gaussian state space model to provide some evidence for time variation in the coefficients of the model. We first focus on the measurement errors, and subsequently extend the discussion to the time-varying regression coefficients.

Figure 5 depicts the evolution of the three volatility components in the measurement equation: The upper panel shows the log-volatilities $h_t$ over time, the middle panel depicts the auxiliary scalings $\tau _t$ used to render the *t*-distribution conditionally Gaussian, and the bottom panel provides the combined volatility series $\tau _t e^{h_t}$ of the measurement errors. The solid black line is the posterior median, the thin black lines indicate the 68 percent posterior coverage interval, while the gray shaded areas refer to National Bureau of Economic Research (NBER) recession dates. The sample starting in January 1927 features 15 distinct periods where the US economy was in recession, with an apparent empirical regularity that recessionary episodes are associated with elevated stock market volatility.

Volatilities in terms of the combined series in the bottom panel peak early in the sample during the Great Depression ranging from the end of 1929 to early 1933. The second-largest peak occurs during the Recession of 1937–1938 which is usually considered minor to the Great Depression, even though it is among the worst recessions over the time span considered. For comparison, volatilities during this period reached levels almost twice as high as during the great financial crisis and the Great Recession from late 2007 to mid-2009. A further notable recessionary episode is the 1973 oil crisis coupled with the 1973–1974 stock market crash, prominently discussed in the context of forecasting excess returns in Welch and Goyal (2008).

Apart from high-volatility episodes during recessions, some further stock market-related events are worth mentioning. Figure 5 clearly shows the so-called Kennedy Slide of 1962, one of the first significant high-volatility periods after World War II, with large stock market declines. Moreover, the volatility series feature the famous Black Monday in October 1987, associated with the greatest one-day percentage decline in US stock market history. The Russian crisis and related collapse of the hedge fund Long-Term Capital Management in the late 1990s is visible, followed by a period of elevated volatilities prior to the burst of the Dot-com bubble. An interesting observation is that such idiosyncratic events typically result in high frequency peaks in terms of $\tau _t$, indicating the necessity of a heavy-tailed error distribution to adequately address such shocks.

We now turn to the time-varying regression coefficients associated with stock fundamentals, depicted in Fig. 6. The solid black line indicates the posterior median,^9^ while the red line marks zero. We omit indicators for NBER recessions for better readability, based on the notion that shifts in coefficients do not appear to be systematically related to distinct stages of the business cycle. The dynamic evolution of the series can be classified into three categories: First, some coefficients are approximately shrunk toward constancy. This class contains NTIS, TBL, LTR, and given the scale of the respective coefficient, also DY. Second, we obtain parameters that strictly decrease or increase over the sample period. Variables roughly featuring such coefficients are the intercept, SVAR, BM, LTY, DFY, DFR and INFL. Third, for DP and EP, we observe coefficients of varying magnitude at different points in time. The paths of both states appear similar, with initial coefficients close to zero gradually gaining importance with peaks around 1980 and subsequent declines. Abrupt shifts governed by the *t*-distributed state equation errors mainly occur in the context of DP and EP.

The dynamics regarding the importance of covariates observed in Fig. 6 are roughly in line with the findings in Welch and Goyal (2008), who estimate a set of models featuring different subsets of the variables and evaluate in-sample fit and out-of-sample forecast performance over time. Our study differs in the sense that the model includes all variables at once (labeled kitchen-sink regression in their study), and stochastically selects both inclusion and exclusion of quantities, besides whether variables differ in importance over time.

## Footnotes

1 For more recent related studies, see, e.g., Pettenuzzo et al. (2014), Nonejad (2017), Ghysels et al. (2018), Gu et al. (2020), Tsiakas et al. (2020), Lansing et al. (2022), or the overviews given in Rapach and Zhou (2013) and Timmermann (2018).

2 With S &P 500 excess returns, we mean those of the aggregate index. These data are available at a number of different frequencies and at different levels of aggregation (e.g., individual stocks, industries, etc.). For the purpose of this paper, limited by the availability of higher-frequency covariates, we opt for monthly observations. We leave applications of our model to other settings and data frequencies, e.g., in the context of trading strategies as in Papaioannou et al. (2017), for future research.

3 In this paper, we consider the model in its non-centered parameterization with no correlation structure between the latent states to provide a straight-forward framework to introduce shrinkage. A possible fruitful avenue for future research would be to introduce a multivariate *t*-distribution for the law of motion of the latent states.

4 For a theoretical discussion of this choice, see Bhattacharya et al. (2015).

5 For recent applications of shrinkage priors within the class of state space models, see, e.g., Bitto and Frühwirth-Schnatter (2019) and Huber et al. (2021).

6 To avoid draws that imply infinite conditional variance or “almost-Gaussianity,” we furthermore restrict the degrees of freedom to the interval [2, 50]. This particular choice, however, shows almost no influence on the results reported in Sect. 3.

7 We rely on this sampling period to allow for one-to-one comparisons with Welch and Goyal (2008).

8 An interesting alternative specification could be to assess whether a finite number of regimes, e.g., recessions/expansions may provide a fruitful competing model, see also Tsiakas et al. (2020). We leave this aspect in the context of our proposed approach for future research.
